# Central Texas Medical Association—Election of Officers, Etc.

**Published:** 1889-01

**Authors:** 


**CENTRAL TEXAS MEDICAL ASSOCIATION—ELECTION OF OFFICERS, ETC.**

This flourishing organization held its regular quarterly session at Waco, Jauuary 8. There was a good attendance.

A paper on "Gynecological practice" was read by Dr. S. M. Hunter of Moody; one on "tobacco as a prophylactic in tuberculosis," by Dr. J. W. Cock, of Waco; "albuminuria in pregnancy," by Dr. Denman, of Bell county.

Dr. H. C. Ghent, of Belton, ex-president Texas State Medical Association, was elected president for the ensuing year; Dr. P. M. Kuykendall, of Moody, first vice president; Dr. W. C. Blalock, of Kosse, second vice president. Dr. J. B. Robertson, of Goliad; third vice president.

Dr. W. O. Wilkes, son of Dr. W. H. Wilkes, of Waco, secretary.

Dr. R. E. Brown, of Waco, son of Dr. H. W. Brown, corresponding secretary, and Dr. S. E. Shelton, of Waco, treasurer.

The meeting closed with a sumptuous banquet. We are pleased to note the integrity and growth of this influential and working body. Next meeting April 9.

